# Preparation and Performance Exploration of MoS_2_/WSe_2_ Van Der Waals Heterojunction Tunneling Field-Effect Transistor

**DOI:** 10.3390/mi16101108

**Published:** 2025-09-29

**Authors:** Chen Chong, Hongxia Liu, Shulong Wang, Shupeng Chen, Cong Yan

**Affiliations:** Key Laboratory for Wide-Band Gap Semiconductor Materials and Devices of Education, The School of Microelectronics, Xidian University, Xi’an 710071, China; zhongchen@xidian.edu.cn (C.C.); slwang@xidian.edu.cn (S.W.); chenshupeng999@126.com (S.C.); ycong16@163.com (C.Y.)

**Keywords:** TMDs (transition metal dichalcogenides), TFET (tunneling field-effect transistor), van der Waals heterojunction

## Abstract

Due to their high carrier mobility, thermal conductivity, and exceptional foldability, transition metal dichalcogenides (TMDs) present promising prospects in the realm of flexible semiconductor devices. Concurrently, tunneling field-effect transistors (TFETs) have garnered significant attention owing to their low energy consumption. This study investigates a TMD van der Waals heterojunction (VdWH) TFET, specifically by fabricating MoS_2_ field-effect transistors (FETs), WSe_2_ FETs, and MoS_2_/WSe_2_ VdWH TFETs. The N-type characteristics of the MoS_2_ and P-type characteristics of WSe_2_ are established through an analysis of the electrical characteristics of the respective FETs. Finally, we analyze the energy band and electrical characteristics of the MoS_2_/WSe_2_ VdWH TFET, which exhibits a drain current switching ratio of 10^5^. This study provides valuable insights for the development of novel low-power devices.

## 1. Introduction

As the manufacturing process of semiconductor devices approaches its physical limits, issues related to power consumption and heat dissipation are becoming increasingly severe. Consequently, Moore’s Law is gradually becoming obsolete [1,2,3]. In response, researchers have begun to explore alternatives for the post-Moore era, with notable devices including TFETs [4,5], negative capacitance FETs [6,7], and two-dimensional (2D) material transistors [8,9,10,11]. Transition metal dichalcogenides (TMDs), characterized by their 2D layered structure similar to graphene, have garnered significant attention from both domestic and international scholars [12]. The layers of TMDs are held together by van der Waals forces, allowing for the detachment of single or multiple layers from the bulk material. Unlike graphene, which possesses a zero-band-gap structure, TMDs feature an adjustable band gap, resulting in unique optical and electrical properties [13,14,15,16]. Additionally, TMDs can be combined with various 2D materials to create heterojunctions, with minimal concerns regarding lattice mismatch [17]. Therefore, TMD-based FETs and TMD-based van der Waals TFETs are highly worthy of research [18].

Numerous simulation studies have been conducted on transition metal dichalcogenide (TMD) field-effect transistors (FETs) and TMD van der Waals heterostructure (vdWH) FETs [19,20,21]. For instance, Qianwen Wang employed the ATK simulator to theoretically investigate the effects of biaxial tensile strain on the transport characteristics of monolayer WSe_2_ double-gate tunneling FETs (DG-TFETs). The findings indicate that increased strain results in a lower subthreshold swing (SS); notably, at 2% strain, the enhancement in the on-state current and maximum transconductance is maximized [22]. Similarly, Fan Chen examined the interlayer tunneling FET of MoS_2_-WTe_2_, revealing that the SS of the MoS_2_-WTe_2_ interlayer TFET is below 60 mV/dec. Furthermore, the optimized device can achieve a drain current of 1000 µA/µm at a supply voltage (VDD) of 0.3 V [23]. To explore the transport mechanisms in vdWH FETs, A. Afzalian integrated density functional theory (DFT) with the non-equilibrium Green’s function (NEGF) method within the atomic modeling solver (ATOMOS). This approach has also been applied to investigate the transport properties of HfS_2_/WSe_2_ TFETs and WTe_2_/WS_2_ vdWH FETs, both exhibiting high on-state currents [24].

Recent experimental studies on field-effect transistors (FETs) utilizing transition metal dichalcogenides (TMDs) and their van der Waals heterostructures (Vdwh FETs) have reported heterogeneous device performances and operational mechanisms [25,26,27,28,29,30]. For instance, Han et al. (2019) fabricated a MoS_2_ FET with material synthesized via atmospheric pressure chemical vapor deposition (APCVD), reporting an on-state current of 2.75 × 10^−1^ µA/µm and an off-state current of 10^−6^ µA/µm at a drain voltage of 0.9 V [31]. Mbisike et al. (2022) integrated Ta_2_O_5_ as a gate dielectric in a WSe_2_ FET, observing p-type transfer characteristics alongside n-type output behavior, with an on-state current of 165 nA at V_DS_ = 1 V [32]. In 2023, Arun Kumar et al. demonstrated that the on-state current in a MoS_2_ FET scales linearly with optical illumination intensity, further establishing that gate bias and light collectively modulate the charge storage states in MoS_2_-based optoelectronic memory [33].

In this work, we experimentally fabricated and characterized back-gated MoS_2_ FETs, WSe_2_ FETs, and a MoS_2_/WSe_2_ van der Waals heterojunction tunnel field-effect transistor (Vdwh TFET). Electrical characterization of the homogeneous devices facilitated polarity determination and informed subsequent analysis of the heterojunction device. Section 2 outlines the material synthesis and device fabrication processes. Section 3 presents a detailed theoretical and experimental analysis of the electronic transport properties in each device, culminating in a band-alignment-based interpretation of the heterojunction TFET operation. Section 4 summarizes the results.

## 2. Material Synthesis and Device Fabrication

The MoS_2_/WSe_2_ heterostructure was fabricated by employing a multi-step lithographic and dry-transfer process:

(a) Photolithographic patterning was performed on a p^+^-Si substrate with 300 nm thermal oxide to define alignment markers.
(b) Few-layer MoS_2_ flakes were mechanically exfoliated from bulk crystal and precisely transferred onto designated coordinates using a dry-transfer technique.
(c) Similarly, WSe_2_ flakes were exfoliated onto a polydimethylsiloxane (PDMS) elastomer stamp.
(d) Using a micro-manipulator under an optical microscope, WSe_2_ was aligned and transferred onto the pre-placed MoS_2_ flake through thermal release to form an intimate van der Waals heterojunction.
(e) Electron-beam lithography followed by electron-beam evaporation of Cr/Au (10/30 nm) was used to define electrical contacts.

**Figure 1:**
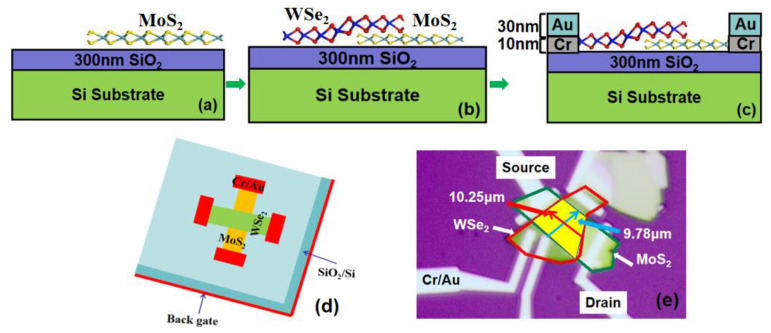
(**a**–**c**) Preparation process of MoS_2_/WSe_2_ Vdwh TFET, (**d**) schematic diagram, and (**e**) OM image of the MoS_2_/WSe_2_ heterojunction.

Figure 1a–c schematically illustrates the fabrication sequence. Maximization of the overlap between MoS_2_ and WSe_2_ flakes is critical for achieving clean heterointerfaces and minimizing interfacial contaminants, as evidenced in the optical micrograph presented in Figure 1e. In the fabricated device, MoS_2_ serves as the drain, while WSe_2_ functions as the source. The dimensions of the heterostructure—length, width, and estimated area—are denoted as 10.25 µm, 9.78 µm, and 100.35 µm^2^, respectively.

Electrical characterization was conducted using an Agilent B1500A semiconductor parameter analyzer under dark conditions at 300 K to minimize environmental noise and optoelectric effects. The system configuration emphasized high-resolution measurements. The measurement system employed two B1517A High-Resolution Source Measure Units (HRSMUs) to bias the gate and drain terminals, respectively, while the source electrode was grounded via an MPSMU. The instrument provides a current resolution of 0.1 fA and a voltage resolution of 100 nV. The range mode was set to auto-ranging to maintain optimal accuracy across the entire sweep. The number of power line cycles (NPLC) was configured between 2 and 5 to achieve a balance between measurement precision and acquisition speed; higher NPLC values were selected for low-current measurements to enhance accuracy. Additionally, a moving-average filter with a window of 8 to 16 points was applied to effectively suppress random noise.

**Figure 2:**
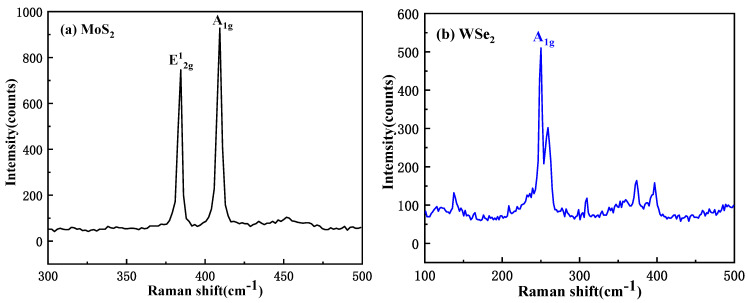
Raman spectrum distribution of the (**a**) MoS_2_ and (**b**) WSe_2_.

Layer thicknesses of MoS_2_ and WSe_2_ were confirmed via Raman spectroscopy. The frequency difference (Δk ≈ 24.855 cm^−1^) between the E^1^_2_g and A_1_g peaks (Figure 2a) confirms trilayer MoS_2_. The A_1_g peak position and the characteristic feature at ~310 cm^−1^ in Figure 2b indicate a multilayer WSe_2_ flake.

## 3. Results and Discussion

To conduct a detailed study on the performance of the MoS_2_/WSe_2_ Vdwh TFET, the electrical characteristics of the MoS_2_ FET and WSe_2_ FET were analyzed first.

### 3.1. MoS_2_ FET

**Figure 3:**
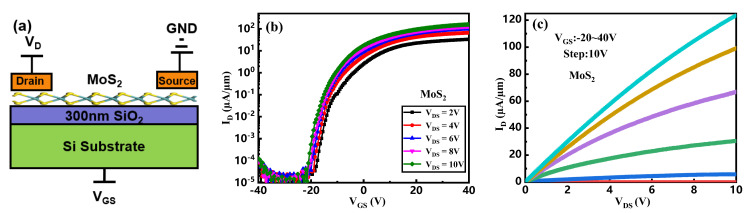
(**a**) Schematic diagram of MoS_2_ FET, (**b**) transfer curve, and (**c**) output curve of MoS_2_ FET.

Figure 3a illustrates the schematic structure of a MoS_2_ FET, in which a positive bias is applied to the drain terminal while the source is grounded. The transfer and output characteristics of the device are presented in Figure 3b and Figure 3c, respectively. As evidenced by the transfer curve in Figure 3b, the MoS_2_ FET operates as an n-channel depletion-mode transistor, indicating that the channel is already conductive at zero gate voltage. The device exhibits an on-state current of 157 µA/µm and an off-state current of 6.5 × 10^−6^ µA/µm, yielding a current switching ratio of 2.4 × 10^8^—a value indicative of high performance in switching applications.

From the output characteristics in Figure 3c, it can be observed that even at V_DS_ = 10 V and V_GS_ = 40 V, the current does not exhibit clear saturation. This suggests that a further increase in V_DS_ under the same gate bias could lead to additional output current enhancement. Such behavior is consistent with the electrostatic properties of two-dimensional materials, where the absence of drain-induced barrier lowering (DIBL) allows the application of high drain voltages without significant short-channel effects, thereby supporting high current drive.

### 3.2. WSe_2_ FET

**Figure 4:**
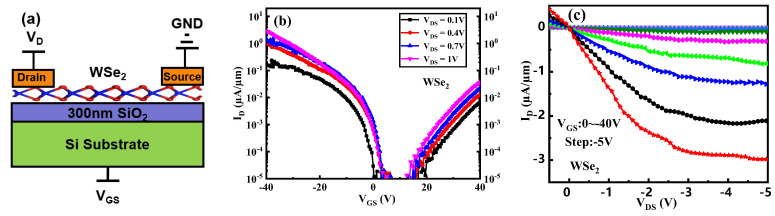
(**a**) Schematic diagram of WSe_2_ FET, (**b**) transfer curve, and (**c**) output curve of WSe_2_ FET.

Figure 4a presents a schematic illustration of a WSe_2_ FET, with a positive bias applied to the drain and the source terminal grounded. The corresponding transfer and output characteristics are displayed in Figure 4b and Figure 4c, respectively. As evidenced by the transfer curve in Figure 4b, the WSe_2_ FET exhibits ambipolar conduction behavior. Specifically, the device operates as an n-channel enhancement-mode FET when the gate voltage exceeds 10 V and as a p-channel depletion-mode FET when the gate voltage is below 10 V.

Notably, the on-state current of the p-type regime reaches 2.9 µA/µm, which is two orders of magnitude higher than that of the n-type regime (3.6 × 10^−2^ µA/µm)—a significant disparity frequently observed in two-dimensional FETs. Given the more pronounced p-type conduction characteristics, this study focuses primarily on the p-type operation of the WSe_2_ FET.

Figure 4c depicts the output characteristics of the WSe_2_ FET under various negative gate voltages. Owing to the unencapsulated device structure, fluctuations observed in the output curves are attributable to unstable contact resistance during electrical measurement. At V_DS_ = −5 V and V_GS_ = −40 V, the output current reaches saturation, with a saturation current of −2.26 µA/µm.

### 3.3. MoS_2_/WSe_2_ Vdwh TFET

**Figure 5:**
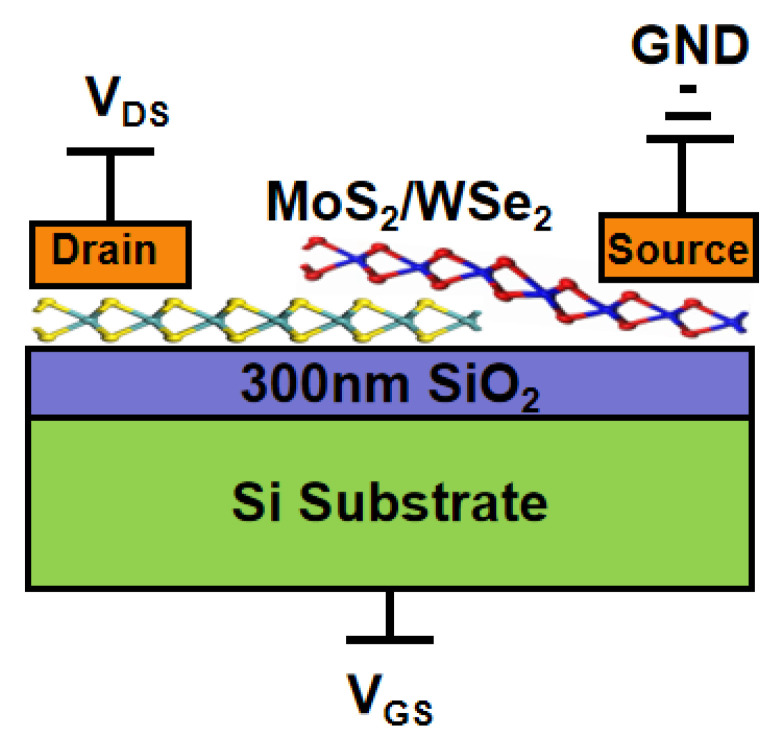
Schematic diagram of MoS_2_/WSe_2_ Vdwh TFET.

Based on the aforementioned electrical characterization, MoS_2_ is identified as an n-type semiconductor, while WSe_2_ exhibits predominant p-type characteristics. Accordingly, in the TFET configuration, WSe_2_ was designated as the source and MoS_2_ as the drain, with the MoS_2_/WSe_2_ van der Waals heterojunction serving as the channel region. A schematic of the resulting MoS_2_/WSe_2_ VdWh TFET is presented in Figure 5, illustrating the architecture in which a positive bias is applied to the MoS_2_ drain and the WSe_2_ source is grounded.

**Figure 6:**
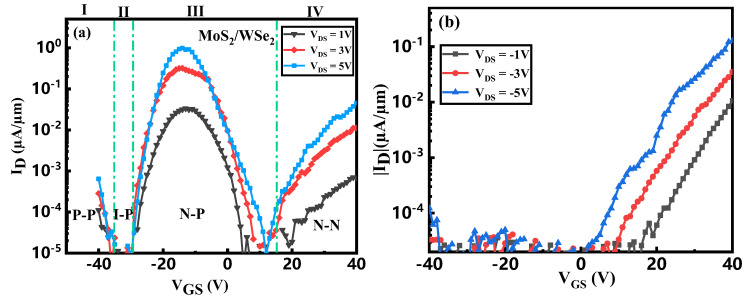
Transfer curve of a MoS_2_/WSe_2_ Vdwh TFET with (**a**) positive drain voltage and (**b**) negative drain voltage.

As illustrated in Figure 6a, the transfer characteristics of the MoS_2_/WSe_2_ van der Waals heterojunction TFET under positive drain bias can be categorized into four distinct operational regimes:

- For V_GS_ < −35 V, the device operates in a P–P state, characterized by strong hole accumulation in WSe_2_ and weak hole accumulation in MoS_2_.
- Within the range of −35 V < V_GS_ < −30 V, the device enters an I–P state, where WSe_2_ maintains strong hole accumulation while MoS_2_ remains in the off-state.
- Over the interval −30 V < V_GS_ < 15 V, an N–P state emerges, featuring strong hole accumulation in WSe_2_ and pronounced electron accumulation in MoS_2_. This regime further divides into two sub-regions: the heterostructure exhibits n-channel FET behavior for −30 V < V_GS_ < −14 V and p-channel FET behavior for −14 V < V_GS_ < 15 V.
- For V_GS_ > 15 V, the device operates in an N–N state, with strong electron accumulation in MoS_2_ and weak electron accumulation in WSe_2_.

As shown in Figure 6b, under negative drain bias, the device exhibits negative conduction, which is that the drain current becomes negative upon application of a positive gate voltage. The transfer curve does not reach saturation even at V_GS_ = 40 V and V_DS_ = −5 V, indicating that the transistor can sustain higher gate and drain voltages without performance degradation.

In TFET devices, the calculation formula of carrier tunneling probability *T_WKB_* is as follows [34]:

(1) $$T_{WKB}=exp\left(-\frac{4\lambda \sqrt{2m^{*}}\sqrt{E_{g}^{3}}}{3q\hbar \left(E_{g}+\Delta \Phi \right)}\right)$$

where m* denotes the effective carrier mass, Δ*Φ* represents the energy difference across the tunnel junction, *E_g_* is the band gap of the semiconductor, and λ corresponds to the effective tunneling length. From Equation (1), it can be deduced that the tunneling probability can be enhanced by increasing Δ*Φ* or reducing λ.

**Figure 7:**
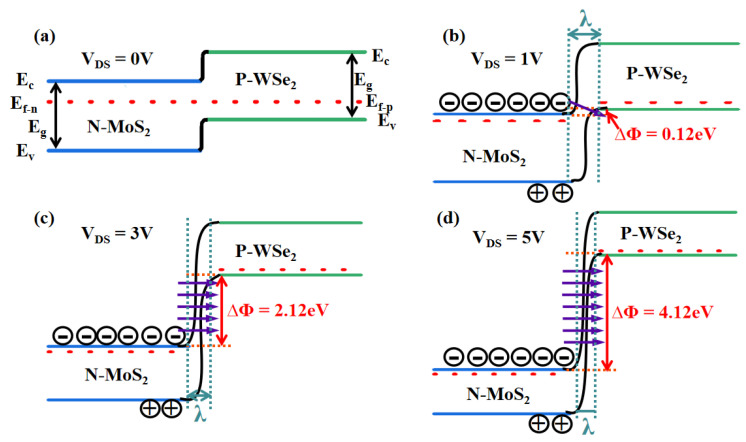
The band-bending diagram of the MoS_2_/WSe_2_ Vdwh at drain voltages of (**a**) 0 V, (**b**) 1 V, (**c**) 3 V, and (**d**) 5 V.

Figure 7 illustrates the energy band diagrams under various drain voltages while the gate voltage is held at 0 V. As evidenced by Figure 7a, the MoS_2_/WSe_2_ heterojunction forms a type-II staggered-gap heterostructure [35]. It can be inferred that as the drain voltage increases, both the enlargement of Δ*Φ* and the reduction in λ contribute to an increase in carrier tunneling probability. Consequently, the tunneling current rises accordingly with drain voltage, a trend that aligns with the behavior observed in Figure 6a.

**Figure 8:**
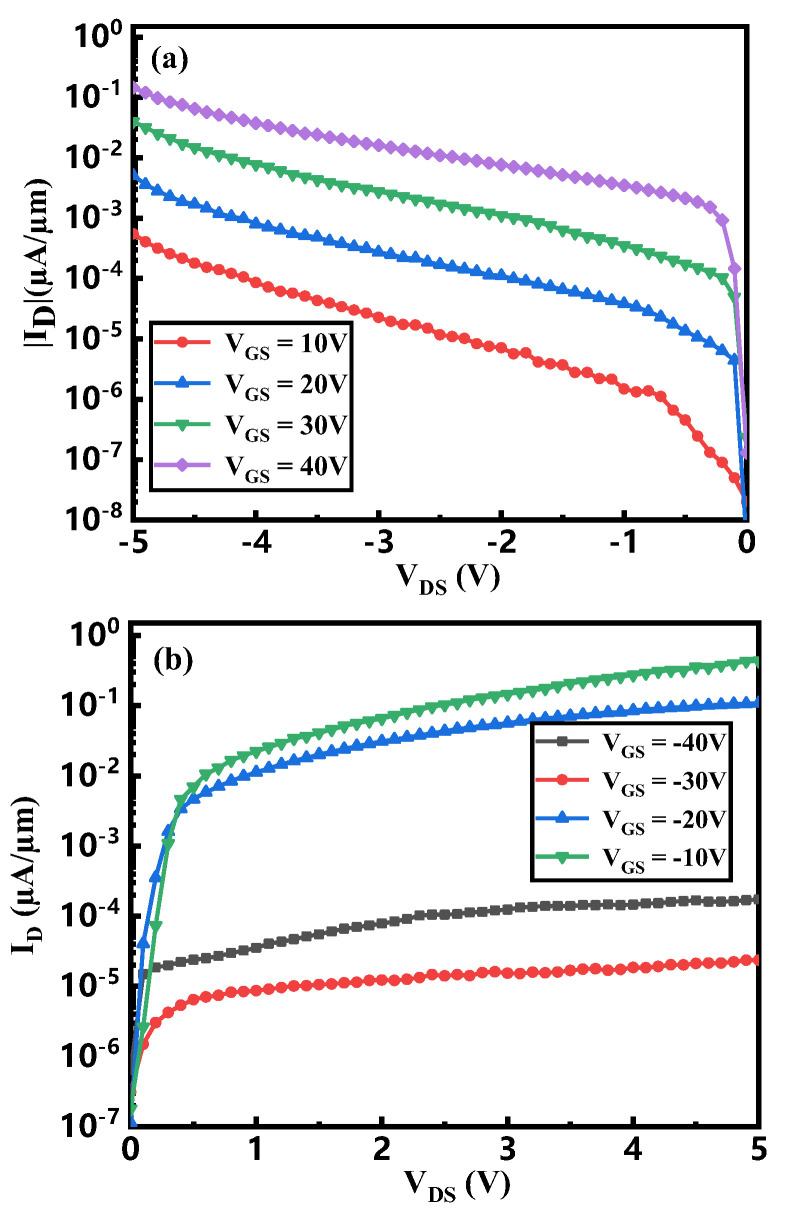
Output curve of a MoS_2_/WSe_2_ Vdwh TFET with (**a**) positive gate voltage and (**b**) negative gate voltage.

As depicted in Figure 8, the output characteristics are separately plotted under negative and positive gate voltages to facilitate a clearer comparison of the device’s operational regimes under different bias conditions. Figure 8a indicates that under negative drain voltage, the saturation drain current increases with more positive gate bias, reaching a maximum value of 20 µA/µm. In contrast, under positive drain bias (Figure 8b), the saturation current ranges from 2.35 × 10^−5^ µA/µm at V_GS_ = −30 V to 4.33 × 10^−2^ µA/µm at V_GS_ = −10 V. No monotonic correlation between the saturation current and gate voltage is observed in this regime.

At V_GS_ = −30 V, the device operates in the I–P state, wherein MoS_2_ is completely turned off, resulting in a significantly reduced drain current. In comparison, at VGS = −10 V, the heterojunction operates in the N–P state, with both WSe_2_ and MoS_2_ conducting, yielding a higher drain current of 0.99 µA/µm, as illustrated in the corresponding region of Figure 8a.

### 3.4. Comparison of Key Parameters of Four Different 2Dl FETs

In this study, three types of two-dimensional FETs were fabricated and characterized, namely, a MoS_2_ FET, WSe_2_ FET, and MoS_2_/WSe_2_ van der Waals heterojunction (Vdwh) TFET. Both the WSe_2_ FET and the MoS_2_/WSe_2_ Vdwh TFET demonstrate configurable polarity through the application of different gate and drain voltages. Accordingly, this section presents a comparative analysis of the electrical properties of four distinct transistor configurations: MoS_2_ nFET, WSe_2_ pFET, MoS_2_/WSe_2_ nTFET, and MoS_2_/WSe_2_ pTFET.

In FETs based on two-dimensional materials, the carrier saturation mobility (*μ_sat_*) is a critical parameter that governs the on-state current and switching speed, thus necessitating accurate extraction. The saturation mobility can be derived from the saturation drain current expression in the transfer characteristics [36], as given by

(2) $$I_{D}=\mu_{sat}C_{g}\frac{W}{2L}{\left(V_{GS}-V_{th}\right)}^{2}\;$$

where *I_D_* denotes the drain current, *μ_sat_* the saturation mobility, *C_g_* the gate capacitance per unit area (for 300 nm SiO_2_, *C_g_* = 11.5 nF·cm^−2^), *V_GS_* the gate voltage, *V_th_* the threshold voltage, and *W* and *L* the channel width and length, respectively.

The key electrical parameters of the various 2D FETs are summarized in Table 1. The MoS_2_ NFET exhibits a high saturation electron mobility of 26 cm^2^·V^−1^·s^−1^ and a subthreshold swing (SS) of 3.28 V/dec, indicating superior switching performance. In contrast, the WSe_2_ PFET shows a modest hole mobility of 0.34 cm^2^·V^−1^·s^−1^ and an SS of 4.65 V/dec, reflecting its relatively limited switching response. The MoS_2_/WSe_2_ nTFET and PTFET inherit the low off-state current from the MoS_2_ NFET and the low on-state current characteristic of the WSe_2_ PFET. As a result, their carrier saturation mobilities are substantially reduced—though still approximately double that of the WSe_2_ PFET—while maintaining comparable SS values.

## 4. Conclusions

In this paper, three types of two-dimensional field-effect transistors (2D FETs)—namely, a MoS_2_ FET, WSe_2_ FET, and MoS_2_/WSe_2_ van der Waals heterojunction (Vdwh) TFET—were fabricated and experimentally characterized. The electrical performance of these devices was evaluated through detailed analysis of their transfer and output characteristics. The MoS_2_ NFET demonstrated a high saturation electron mobility of 26 cm^2^·V^−1^·s^−1^ and a current on/off ratio of 2.4 × 10^8^ at a drain voltage of 10 V. Meanwhile, the MoS_2_/WSe_2_ NTFET, measured at a drain voltage of 5 V, exhibited a saturation electron mobility of 0.8 cm^2^·V^−1^·s^−1^ and a current on/off ratio of 1.15 × 10^5^. The methodology employed in this work—analyzing heterojunction TFET performance through constituent single-material FETs—offers a valuable strategy for the investigation of 2D heterojunction tunnel devices. This approach not only provides insights into the design and optimization of such structures but also facilitates the development of low-power electronic devices based on two-dimensional materials.

## Figures and Tables

**Table 1:** Comparison of the electrical characteristic parameters of the different FETs.

FET	*µ_sat_* (cm^2^ V^−1^ s^−1^)	Vth (V)	SS (V/Dec)	I_on_/I_off_	I_on_ (µA/µm)	I_off_ (µA/µm)
MoS_2_ NFET	26 ± 2	−21~−18	3.3	2.4 × 10^8^	157	6.5 × 10^−6^
WSe_2_ PFET	0.3 ± 0.1	0~5	4.7	2.9 × 10^4^	2.9	1 × 10^−4^
MoS_2_/WSe_2_ NTFET	0.8 ± 0.1	−32~−30	4.0	1.15 × 10^5^	0.99	8.6 × 10^−6^
MoS_2_/WSe_2_ PTFET	0.5 ± 0.2	5~10	5.2	1.03 × 10^5^	0.99	9.6 × 10^−6^

## Data Availability

The original contributions presented in the study are included in the article, further inquiries can be directed to the corresponding author.

## References

[1] Ieong M., Doris B., Kedzierski J., Rim K., Yang M. (2004). Silicon device scaling to the sub-10-nm regime. Science.

[2] Hiblot G., Dutta T., Rafhay Q., Lacord J., Akbal M., Boeuf F., Ghibaudo G. (2015). Accurate Boundary Condition for Short-Channel Effect Compact Modeling in MOS Devices. IEEE Trans. Electron Devices.

[3] Chander S., Sinha S.K., Chaudhary R. (2022). Comprehensive review on electrical noise analysis of TFET structures. Superlattices Microstruct..

[4] Nasani K., Bhowmick B., Pukhrambam P.D. (2023). Effect of lateral straggle parameter on Hetero Junction Dual Gate Vertical TFET. Microelectron. J..

[5] Gorla S.R.K., Pandey C.K. (2024). Reduced OFF-state current and suppressed ambipolarity in a dopingless vertical TFET with dual-drain for high-frequency circuit applications. AEU-Int. J. Electron. Commun..

[6] Malvika, Choudhuri B., Mummaneni K. (2022). A new pocket-doped NCFET for low power applications: Impact of ferroelectric and oxide thickness on its performance. Micro Nanostruct..

[7] Kaushal S. (2023). Introduction to Newly Adopted NCFET and Ferroelectrics for Low-Power Application. Advanced Ultra Low-Power Semiconductor Devices: Design and Applications.

[8] Giubileo F., Di Bartolomeo A. (2017). The role of contact resistance in graphene field-effect devices. Prog. Surf. Sci..

[9] Geim A., Grigorieva I. (2013). Van der Waals heterostructures. Nature.

[10] Cao W., Kang J., Sarkar D., Liu W., Banerjee K. (2015). 2D semiconductor FETs—Projections and design for sub-10 nm VLSI. IEEE Trans. Electron Devices.

[11] Berahman M., Aghasi H. (2025). Tunneling Field Effect Transistors Based on Janus Monolayer PtSSe. IEEE Trans. Nanotechnol..

[12] Wang Q.H., Kalantar-Zadeh K., Kis A., Coleman J.N., Strano M.S. (2012). Electronics and optoelectronics of two-dimensional transition metal dichalcogenides. Nat. Nanotechnol..

[13] Liu L., Liu C., Huang X., Zeng S., Tang Z., Zhang D.W., Zhou P. (2022). Tunable Current Regulative Diode Based on Van der Waals Stacked MoS_2_/WSe_2_ Heterojunction–Channel Field-Effect Transistor. Adv. Electron. Mater..

[14] Nourbakhsh A., Zubair A., Dresselhaus M.S., Palacios T. (2016). Transport properties of a MoS_2_/WSe_2_ heterojunction transistor and its potential for application. Nano Lett..

[15] Zhao J., Li N., Yu H., Wei Z., Liao M., Chen P., Wang S., Shi D., Sun Q., Zhang G. (2017). Highly Sensitive MoS_2_ Humidity Sensors Array for Noncontact Sensation. Adv. Mater..

[16] Di Bartolomeo A., Kumar A., Durante O., Sessa A., Faella E., Viscardi L., Intonti K., Giubileo F., Martucciello N., Romano P. (2023). Temperature-dependent photoconductivity in two-dimensional MoS_2_ transistors. Mater. Today Nano.

[17] Chava P., Kateel V., Watanabe K., Taniguchi T., Helm M., Mikolajick T., Erbe A. (2024). Electrical characterization of multi-gated WSe_2_/MoS_2_ van der Waals heterojunctions. Sci. Rep..

[18] Yang M., Lu Y., Zhang Q., Han Z., Zhang Y., Liu M., Zhang N., Hu H., Wang L. (2022). Charge transport behaviors in a multi-gated WSe_2_/MoS_2_ heterojunction. Appl. Phys. Lett..

[19] Silvestri L., Palsgaard M., Rhyner R., Frey M., Wellendorff J., Smidstrup S., Gull R., El Sayed K. (2023). Hierarchical modeling for TCAD simulation of short-channel 2D material-based FETs. Solid-State Electron..

[20] Mounir A., Iñiguez B., Lime F., Kloes A., Knobloch T., Grasser T. (2023). Compact I-V model for back-gated and double-gated TMD FETs. Solid-State Electron..

[21] Bedia A., Bedia F.Z., Benyoucef B. (2011). 2D Device Modeling and Simulation/FET for Biomedical Applications. Phys. Procedia.

[22] Wang Q., Sang P., Li Y., Chen J. Impacts of Biaxial Tensile Strain in Double-gate Tunneling Field-effect-transistor (DG-TFET) with a Monolayer WSe_2_ Channel. Proceedings of the 2020 IEEE Silicon Nanoelectronics Workshop (SNW).

[23] Chen F., Ilatikhameneh H., Tan Y., Valencia D., Klimeck G., Rahman R. (2016). Transport in vertically stacked hetero-structures from 2D materials. J. Phys. Conf. Ser..

[24] Afzalian A., Akhoundi E., Gaddemane G., Duflou R., Houssa M. (2021). Advanced DFT–NEGF Transport Techniques for Novel 2-D Material and Device Exploration Including HfS_2_/WSe_2_ van der Waals Heterojunction TFET and WTe_2_/WS_2_ Metal/Semiconductor Contact. IEEE Trans. Electron Devices.

[25] Roy T., Tosun M., Cao X., Fang H., Lien D.H., Zhao P., Chen Y., Chuen Y., Guo J., Javey A. (2015). Dual-gated MoS_2_/WSe_2_ van der Waals tunnel diodes and transistors. ACS Nano.

[26] Roy T., Tosun M., Hettick M., Ahn G.H., Hu C., Javey A. (2016). 2D-2D tunneling field-effect transistors using WSe_2_/SnSe_2_ heterostructures. Appl. Phys. Lett..

[27] Kanungo S., Ahmad G., Sahatiya P., Mukhopadhyay A., Chattopadhyay S. (2022). 2D materials-based nanoscale tunneling field effect transistors: Current developments and future prospects. NPJ 2D Mater. Appl..

[28] Si K., Ma J., Lu C., Zhou Y., He C., Yang D., Wang X., Xu X. (2020). A two-dimensional MoS_2_/WSe_2_ van der Waals heterostructure for enhanced photoelectric performance. Appl. Surf. Sci..

[29] Yang S.H., Yao Y.T., Xu Y., Lin C.Y., Chang Y.M., Suen Y.W., Sun H., Lien C., Li W., Lin Y. (2018). Atomically thin van der waals tunnel field-effect transistors and its potential for applications. Nanotechnology.

[30] Sarkar D., Xie X., Liu W., Cao W., Kang J., Gong Y., Kraemer S., Ajayan P.M., Banerjee K. (2015). A subthermionic tunnel field-effect transistor with an atomically thin channel. Nature.

[31] Han T., Liu H., Wang S., Chen S., Xie H., Yang K. (2019). Probing the Field-Effect Transistor with Monolayer MoS_2_ Prepared by APCVD. Nanomaterials.

[32] Mbisike S.C., Tsiamis A., Lomax P., Cheung R. (2022). Anodic tantalum: Fabrication, breakdown characteristics of capacitor and integration with a WSe_2_ field effect transistor. Solid-State Electron..

[33] Kumar A., Faella E., Durante O., Giubileo F., Pelella A., Viscardi L., Intonti K., Sleziona S., Schleberger M., Di Bartolomeo A. (2023). Optoelectronic memory in 2D MoS_2_ field effect transistor. J. Phys. Chem. Solids.

[34] Schenk A., Heiser G. (1997). Modeling and simulation of tunneling through ultra-thin gate dielectrics. J. Appl. Phys..

[35] Oezcelik V.O., Azadani J.G., Yang C., Koester S.J., Low T. (2016). Band Alignment of 2D Semiconductors for Designing Heterostructures with Momentum Space Matching. Phys. Rev. B.

[36] He F., Qin Y., Wan L., Su J., Lin Z., Zhang J., Chang J., Wu J., Hao Y. (2020). Metal oxide heterojunctions for high performance solution grown oxide thin film transistors. Appl. Surf. Sci..

